# A pilot study of function‐based radiation therapy planning for lung cancer using hyperpolarized xenon‐129 ventilation MRI

**DOI:** 10.1002/acm2.13502

**Published:** 2022-01-19

**Authors:** Yi Ding, Lu Yang, Qian Zhou, Jianping Bi, Ying Li, Guoliang Pi, Wei Wei, Desheng Hu, Qiuchen Rao, Haidong Li, Li Zhao, An Liu, Dongsu Du, Xiao Wang, Xin Zhou, Guang Han, Kun Qing

**Affiliations:** ^1^ Department of Radiation Oncology Hubei Cancer Hospital Tongji Medical College Huazhong University of Science and Technology Wuhan China; ^2^ Department of Radiation Oncology Renmin Hospital Wuhan University Wuhan China; ^3^ Key Laboratory of Magnetic Resonance in Biological Systems, State Key Laboratory of Magnetic Resonance and Atomic and Molecular Physics, National Center for Magnetic Resonance in Wuhan, Wuhan Institute of Physics and Mathematics, Innovation Academy for Precision Measurement Science and Technology Wuhan National Laboratory for Optoelectronics Chinese Academy of Sciences Wuhan China; ^4^ Department of Biomedical Engineering Zhejiang University Hangzhou China; ^5^ Department of Radiation Oncology City of Hope National Medical Center Duarte California USA; ^6^ Department of Radiation Oncology Cancer Institute of New Jersey Rutgers University New Brunswick New Jersey USA

**Keywords:** function‐based radiotherapy treatment planning, hyperpolarized xenon‐129, lung cancer, MRI, RILI, treatment planning

## Abstract

**Purpose:**

Radiation‐induced lung injury (RILI) is a common side effect in patients with non‐small cell lung cancer (NSCLC) treated with radiotherapy. Minimizing irradiation into highly functional areas of the lung may reduce the occurrence of RILI. The aim of this study is to evaluate the feasibility and utility of hyperpolarized xenon‐129 magnetic resonance imaging (MRI), an imaging tool for evaluation of the pulmonary function, to guide radiotherapy planning.

**Methods:**

Ten locally advanced NSCLC patients were recruited. Each patient underwent a simulation computed tomography (CT) scan and hyperpolarized xenon‐129 MRI, then received 64 Gyin 32 fractions for radiotherapy. Clinical contours were drawn on CT. Lung regions with good ventilation were contoured based on the MRI. Two intensity‐modulated radiation therapy plans were made for each patient: an anatomic plan (Plan‐A) based on CT alone and a function‐based plan (Plan‐F) based on CT and MRI results. Compared to Plan‐A, Plan‐F was generated with two additional steps: (1) beam angles were carefully chosen to minimize direct radiation entering well‐ventilated areas, and (2) additional optimization criteria were applied to well‐ventilated areas to minimize dose exposure. V_20Gy_, V_10Gy_, V_5Gy_, and the mean dose in the lung were compared between the two plans.

**Results:**

Plan‐A and Plan‐F were both clinically acceptable and met similar target coverage and organ‐at‐risk constraints (*p* > 0.05) except for the ventilated lungs. Compared with Plan‐A, V_5Gy_ (Plan‐A: 30.7 ± 11.0%, Plan‐F: 27.2 ± 9.3%), V_10Gy_ (Plan‐A: 22.0 ± 8.6%, Plan‐F: 19.3 ± 7.0%), and V_20Gy_ (Plan‐A: 12.5 ± 5.6%, Plan‐F: 11.0 ± 4.1%) for well‐ventilated lung areas were significantly reduced in Plan‐F (*p* < 0.05).

**Conclusion:**

In this pilot study, function‐based radiotherapy planning using hyperpolarized xenon‐129 MRI is demonstrated to be feasible in 10 patients with NSCLC with the potential to reduce radiation exposure in well‐ventilated areas of the lung defined by hyperpolarized xenon‐129 MRI.

## 1 INTRODUCTION

Currently, worldwide lung cancer is the most frequently diagnosed cancer (11.6% of all cases). It is also the leading cause of death from cancer among both men and women, making up 18.4% of all cancer deaths (about 1.7 million) in 2020.^1^ More than half of patients with lung cancer are recommended for radiotherapy including both radiotherapy alone and adjuvant radiotherapy.^2^ Radiation‐induced lung injury (RILI) is a common complication in patients receiving radiation in the lung. It is reported that between 5% and 25% of patients with lung cancer treated by radiotherapy develop pneumonitis, which usually occurs about 4–12 weeks after treatment.^2^, ^3^ More severely, chronic pneumonitis could develop into irreversible lung scarring, called fibrosis, significantly impacting the quality of life for patients. Currently, the exact triggering thresh‐hold for RILI is not well known. The existing clinical practice primarily relies on the volume of lung dose exceeding 20 Gy (V_20_) or 5 Gy (V_5_) and mean lung dose as quantitative metrics to evaluate risks for radiation pneumonitis.^4^, ^5^ Multiple studies were performed to investigate which lung volume on computed tomography (CT) yields the best predictor for the development of radiation pneumonitis, including lung minus planning target volume (PTV), lung minus gross target volume (GTV), ipsilateral, or whole lung.^6^, ^7^ These practices were all based on a simple assumption that lung tissues are homogeneously functioning and respond the same way to radiation toxicity, which may not be accurate. Most lung cancer patients have chronic obstructive pulmonary diseases (COPD),^9^ meaning that the lung function could be spatially heterogeneous. Avoiding high radiation exposure to highly functional lung areas may reduce the likelihood of developing RILI. But how to incorporate the functional information of the lung into radiation therapy planning is an ongoing topic.

The field of functional imaging of the lung is underrapid development in recent years. Single‐photon emission computed tomography (SPECT) imaging is traditionally used to provide functional information of the lung including ventilation using technetium‐99 mor xenon‐133 and perfusion using technetium‐99 m.^10^ Early work by Marks et al.^11^ demonstrated that pulmonary perfusion indicated by SPECT showed unique values in “designing radiation portals to minimize irradiation of functioning lung”. More interestingly, pulmonary perfusion was noticed to decline in 1–6 months in areas receiving 40 Gy of dose. However, relatively low resolution of SPECT (larger than 7 millimeters) limits its utility. CT is dominating in obtaining the anatomical information of the lung. Four‐dimensional CT (4DCT) is also routinely used in radiation oncology to extract information about target or organ movement. Information about ventilation could be obtained from 4DCT using the new technique. The resulting ventilation‐based dose function metrics were found to correlate well with^12^ thoracic toxicity and be a better predictor for^13^, ^14^ RILI after radiation. New imaging techniques such as dual‐energy CT (DECT) using xenon and iodine could also provide ventilation and perfusion information of the lung. However, at the current stage, DECT is not widely available in all clinical sites. Furthermore, the usage of both SPECT and CT is associated with exposure to ionizing radiation, which is not favored for repeated assessment in patients.

Magnetic resonance imaging (MRI) using hyperpolarized (HP) gas such as helium‐3 and xenon‐129 is a promising imaging tool that provides pulmonary functional information of the lung. Both helium‐3 and xenon‐129 are non‐radioactive inert gases. HP helium‐3 MRI has already been approved for clinical use in the United Kingdom (UK). Tahir et al.^15^ investigated the effect of beam angles and field number on functionally guided intensity‐modulated radiotherapy (IMRT) using the ventilation information provided by HP helium‐3 MRI. They found that beam angles have to be properly chosen to avoid highly ventilated areas. Compared to helium‐3, xenon‐129 is naturally abundant and has more stable long‐term availability. More importantly, xenon‐129 has a higher solubility in biological tissues including blood (so‐called dissolved phase), which are differentiable from the gaseous xenon‐129 (gas phase) by MRI. Recent development in MR techniques enables HP MRI using xenon‐129 to provide not only ventilation information of the lung but also unprecedented information about direct gas exchange from the airspace to the blood.^16^, ^17^

The purpose of this study is to investigate the feasibility and potential utility of function‐based treatment planning in patients with lung cancer using the ventilation information provided by HP xenon‐129 MRI.

## 2 MATERIALS AND METHODS

### 2.1 Human subjects

In this study, 10 patients (P1‐P10, age: 46–72 years, male: seven, female: three; median age: 58 years) with locally advanced NSCLC (details in Table 1) were recruited. Inclusion criteria included scheduled for radiation therapy and the ability to hold the breath for 15 s or longer. The study was approved by the institutional review board and written consent has been signed by each participant.

**TABLE 1:** Demographic and clinical information about patients enrolled in this study

**Patient No**.	**Age**	**Gender**	**Histology**	**Stage**	**COPD**	**ECOG score**	**Tumor size (cc)**
P1	60	M	SCC	T3N3M0	N	0	196.3
P2	57	M	SCC	T4N2M0	N	0	150.7
P3	57	F	AC	T2N1M0	N	1	30.3
P4	70	M	SCC	T3N1M0	Y	0	59.1
P5	53	M	AC	T3N0M0	N	0	21.9
P6	64	M	SCC	T2N2M0	N	1	73.6
P7	62	M	SCC	T3N1M0	N	1	53.4
P8	72	F	AC	T3N1M0	Y	1	42.1
P9	46	F	AC	T2N1M0	Y	0	35.3
P10	56	M	SCC	T4N3N0	N	1	234.9

Abbreviations: SCC, squamous cell carcinoma; AC, adenocarcinoma; ECOG score, Eastern Cooperative Oncology Group performance score.

### 2.2 Imaging protocols

#### 2.2.1 Magnetic resonance imaging

MRI was performed on a whole body 1.5 Tesla MR scanner (Avanto, Siemens Medical Solutions, Erlangen, Germany) using the system body coil for proton imaging and a custom‐built transmit‐receiver vest radiofrequency (RF) coil for HP xenon‐129 MRI. Xenon‐129 was polarized with the spin‐exchange optical pumping technique using a commercial polarizer system (verImagin Healthcare, Wuhan, China) to a polarization level of approximately 25%. Patients breathed all the way out and then inhaled 1 L of gas mixture (50% hyperpolarized xenon‐129 + 50% N_2_) for the HP xenon‐129 MRI acquisition. Imaging parameters included TR = 4.2 ms, TE = 1.9 ms, axial field of view = 384 × 192 mm^2^ with an in‐plane resolution of 4 × 4 mm^2^ and slice thickness = 5 mm. A proton MRI was acquired to provide anatomical information about the lung in a separate breath hold. To reproduce the lung inflation level, the patient inhaled 1 L of nitrogen gas with the parameters. Both the HP and proton MR acquisition used spoiled gradient‐echo based pulse sequences. Imaging parameters for the proton MRI included TR = 2.5 ms, TE = 0.8 ms, axial field of view = 384 × 240 mm^2^with in‐plane of 4 × 4 mm^2^ and slice thickness = 5 mm. The acquisition time for xenon‐129 and proton MRI was 12, 9 s, respectively.

#### 2.2.2 Computed tomography

To make the lung volume consistent with MRI acquisition, a CT scan was also performed after 1 L of air was inhaled by each patient. The CT scan was done on a 16‐slice multi‐detector CT scanner (LightSpeed 16, GE Healthcare, Chicago, IL). Scanning parameters used included pitch 1.75: 1, in‐plane resolution = 1 × 1 mm^2^, thickness = 5 mm. CT was acquired on the same day as MRI scans.

### 2.3 Image fusion and target delineation

MRI was fused to CT images using MIM software (MIM software Inc, Beachwood, OH). Registration option “convert local alignments into deformable registration,” which is a combination of rigid and deformable registration was used to align the chest walls. The Jacobian determinant of the registration transform was checked to make sure they are all above zero. GTV, clinical target area (CTV), PTV and organ‐at‐risks (OARs) including lung, heart, spinal cord and esophagus were delineated based on the Radiation Therapy Oncology Group (RTOG) lung cancer delineation guidelines (RTOG 1106). To compensate for the respiratory motion, the PTV was created with 1 cm extension in the superior‐to‐inferior direction, and 5 mm in all other directions from CTV. Lung areas were segmented into four classes based on the signal intensity of the HP xenon‐129 images,^18^ including well‐ventilated, ventilated, hypo‐ventilated and poorly ventilated areas. Lung regions with ventilation signal falling into the first two classes (well‐ventilated and ventilated areas) were defined as ventilated area. The poorly ventilated areas were defined as ventilation defect.

### 2.4 Treatment planning

Treatment planning was performed using the Eclipse planning system (Varian Medical Systems, Palo Alto, CA). All patients were treated to 64 Gyin 32 fractions. Two co‐planar IMRT plans are made for each patient. The routine clinical planning (anatomic plan: Plan‐A) and function‐based planning (Plan‐F) were both set with seven radiation fields, with the isocenter of radiation field set to the geometric center of PTV. The arrangement of the beam angles for Plan‐F was designed to minimize direct radiation entering the well‐ventilated lung areas. Plan‐A was based on planning CT following routine clinical considerations. Planning criteria/parameters for Plan‐A are shown in Table 2. Plan‐F was made with relatively higher priority given to avoid the functional area of the lung. Planning criteria/parameters were the same with Plan‐A, except for lung (Table 2), *D*_mean_ ≤ 15 Gy, V _5Gy_ ≤ 60%, V_10Gy_ ≤ 40%, V_20Gy_ ≤ 28%, V_30Gy_ ≤ 20% were set for ventilated lung area defined by HP MRI instead of the whole lung defined by CT.

**TABLE 2:** Radiation therapy treatment planning goals for Plan‐A and Plan‐F

		**Target and OARs**		**Coverage goals and dose constraints**
Plan‐F	Plan‐A	PTV		V_95%_≥98%, V_100% _> 95%, V110_% _< 15%, V_115% _< 5%
		OARs	Lung	*D*_mean_≤15 Gy, V _5_ _Gy_≤60%, V_10Gy_≤40%, V_20Gy_≤28%, V_30Gy_≤20%
			Esophagus	*D*_max_≤66 Gy; *D*_mean_≤40 Gy
			Spinal cord	*D*_max_≤45 Gy
			Heart	*D*_mean_≤10 Gy, V_20Gy_≤30%, V_30Gy_≤20%
	Plan‐F only		Ventilated lung	*D*_mean_≤15 Gy, V _5_ _Gy_≤60%, V10Gy≤40%, V_20Gy_≤28%, V_30Gy_≤20%

### 2.5 Statistical analysis

Paired *t*‐test was used to compare Plan‐A and Plan‐F in PTV coverage and OAR sparing. A *p*‐value less than 0.05 is considered statistically significant. Analysis was done using SPSS Statistics (Chicago, IL).

## 3 RESULTS

### 3.1 MRI imaging results

**FIGURE 1:**
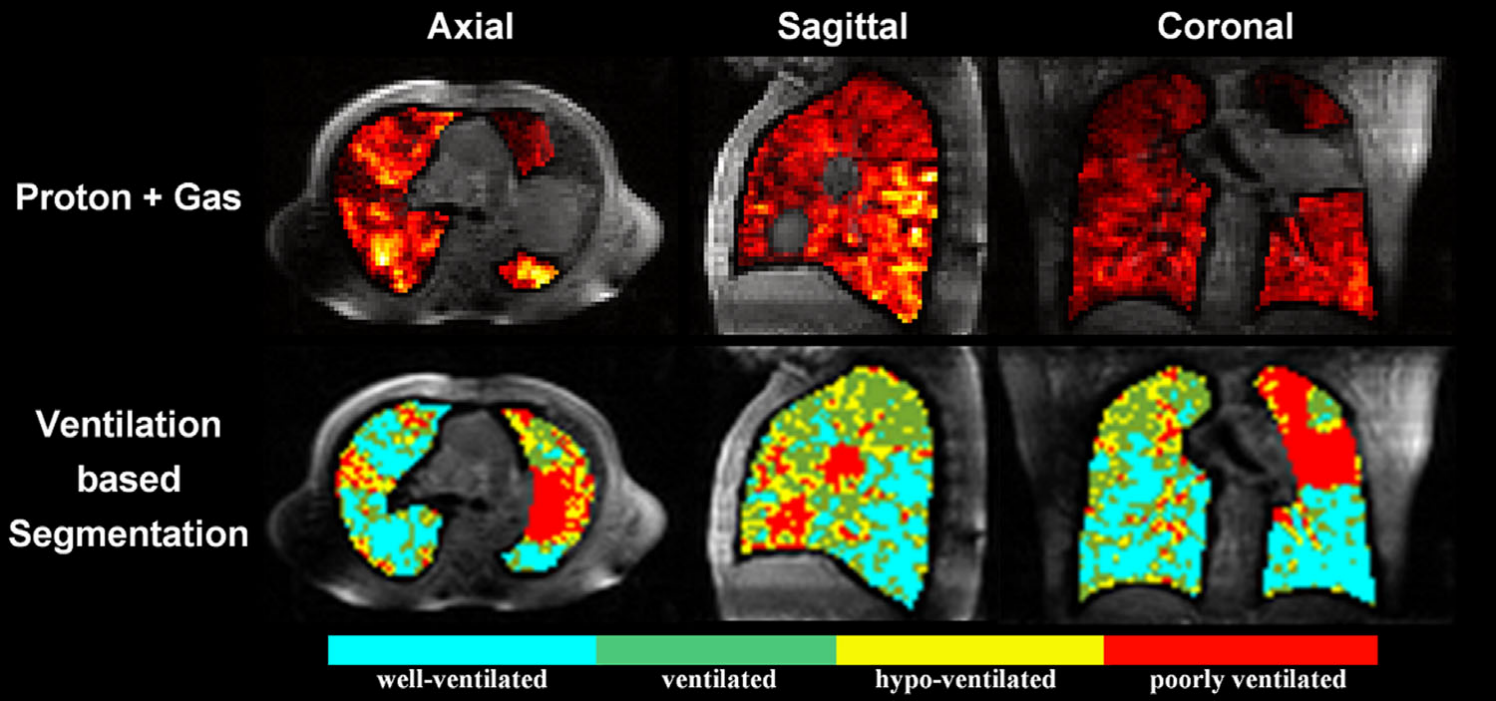
**Upper row**: HP xenon‐129 gas MR images (color) overlaid on proton MR images (grayscale) from patient P1 (Age 60, Male, T3N3M0, Stage III C, AJCC Version 8). Lower row: Segmentation of lung into four clusters based on the signal intensity of HP xenon‐129 gas MR images. The well‐ventilated, ventilated, hypo‐ventilated and poorly ventilated areas are shown in cyan, green, yellow, and red colors, respectively

A three‐dimensional view of representative HP xenon‐129 MR images overlaid on proton MR images from patient P1 is shown in Figure 1 (upper row). The ventilation heterogeneity can be visualized in the images, with bright yellow corresponding to high ventilation, and dark red corresponding to low ventilation. Also shown in the lower row of Figure 1 is a segmentation map of the lung based on the signal intensity of the HP xenon‐129 MR images. The lung was segmented into four clusters corresponding to well‐ventilated (cyan), ventilated (green), hypo‐ventilated (yellow) and poorly ventilated (red) areas. The mean ventilation defect percentage of the whole lung in these ten patients is 9.4 ± 6.2% with relatively large heterogeneity ranging from a minimum of 2.5% and a maximum of 21.6%.

### 3.2 Treatment planning

**FIGURE 2:**
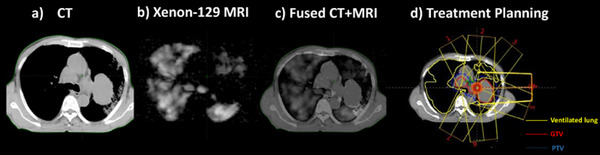
Representative images from patient P1 (Age: 60, Male, T3N3M0, Stage III C, AJCC Version 8). From left to right showing: (a) Simulation CT image for treatment planning; (b) HP xenon‐129 MR images from the corresponding location with the CT image; (c) Fused CT and MRI images; (d) Beam arrangement for radiotherapy treatment planning, with the yellow contours showing the well‐ventilated lung areas

Figure 2 shows a schematic illustrating the treatment‐planning workflow using patient P1 as an example. The planning CT (Figure 2a) and MR images (Figure 2b) were acquired separately. Then the MR images were fused to the CT images (Figure 2c). For treatment planning (Figure 2d), a yellow contour was generated for well‐ventilated lung areas. The selection of beam angles and optimization was determined accordingly.

**FIGURE 3:**
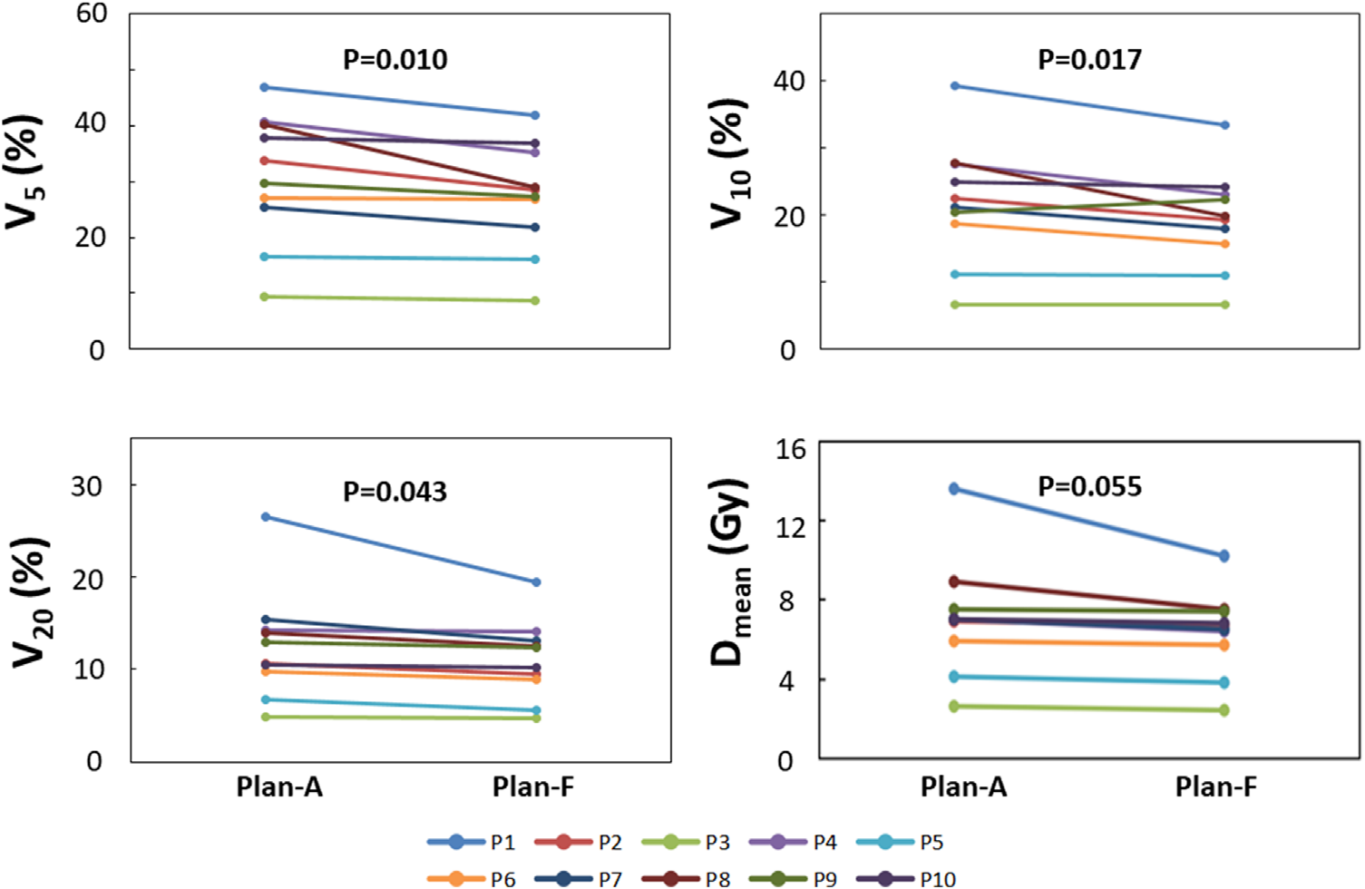
V_5_, V_10_, V_20_ and D_mean_ for a conventional treatment plan (Plan‐A) and functional‐imaging guided treatment plan (Plan‐F) from patients P1‐10. The V_5_, V_10_ and V_20_ were significantly lower in Plan‐F (*p* < 0.05), as compared to those in Plan‐A

Both Plan‐A and Plan‐F met the target coverage goals mentioned above. There was no significant difference in PTV homogeneity index (*p* = 0.34) and conformity index (*p* = 0.82) between Plan‐A and Plan‐F. All plans were judged as clinically acceptable. Similarly, the doses to all OARs except the ventilated areas had no statistical difference (*p* > 0.05). As shown in Figure 3, compared with Plan‐A, the V_5Gy_, V_10Gy_, V_20Gy_ of high‐functional lungs were significantly reduced in Plan‐F (V5: Plan‐A: 30.7 ± 11.0%, Plan‐F: 27.2 ± 9.3%, *p* = 0.010; V10: Plan‐A: 22.0 ± 8.6%, Plan‐F: 19.3 ± 7.0%, *p* = 0.017; V20: Plan‐A: 12.5 ± 5.6%, Plan‐F: 11.0 ± 4.1%, *p* = 0.043;). For the lung mean dose (*D*_mean_), a trend of decrease was in Plan‐F (Plan‐A: 7.0 ± 2.8 Gy, Plan‐F: 6.3 ± 2.0 Gy, *p* = 0.055; Figure 3).

## 4 DISCUSSIONS

In this pilot study, the feasibility and utility of using functional information of the lung to guide radiotherapy planning was investigated in ten patients with NSCLC. This work demonstrated that the treatment planning using ventilated lungs defined by HP xenon‐129 MRI is easily achievable without compromising other planning goals in this cohort of patients. By using static IMRT with appropriate beam arrangement and adjustment of the planning constraints, the dose sparing in the ventilated areas wad significantly reduced (*p* < 0.05 for V_5_, V_20_, etc.) compared to the conventional treatment planning approach only based on CT.

The overall ventilation varied among patients with a minimal ventilation defect of only 2.5% and a maximal ventilation defect of 21.6%. In the optimization, ventilated areas are spared equally. Different optimization strategies may yield different results. More importantly, the spatial ventilation heterogeneity near the treatment target varied substantially. In some patients, relatively high ventilation heterogeneity was observed near the tumor. Figure 2 shows such an example from P1. The strategy used in this study was very effective in improving the DVH metrics for functional lung in these patients, because the selection of beam angles can greatly reduce the high radiation dose exposed to the functional lung near the tumor. However, if the ventilation was relatively homogeneous near the tumor, such as P3, avoiding dose sparing in these areas is not easily achievable.

For radiotherapy application, obtaining functional information of the lung using CT‐based approaches has huge benefits over other imaging modalities. First, CT has superior imaging resolution, geometric integrity, and imaging speed, compared to MRI or SPECT. Second, CT is available in almost every radiation therapy department. For patients with lung cancer, 4DCT is routinely used for contouring and planning. Thus, any functional information extracted from 4DCT is “free” for radiotherapy patients underwent 4DCT for clinical purpose, meaning that they are obtained without any additional cost. However, existing studies showed only moderate correlations between CT‐ventilation and ventilation obtained using other existing imaging modalities. For instance, Kipritidis et al.^19^ compared CT‐ventilation with PET Galli‐gas imaging and found correlations of 0.42 ± 0.16 between them. In a study including 11 patients with lung cancer, Tahir et al.^20^ found CT ventilation correlated only moderately with helium‐3 MRI (*R* = 0.37 ± 0.19) and xenon‐129 MRI (0.33 ± 0.17). An interesting study performed by Castillo et al.^21^ showed dice similarity coefficients of 0.78 between CT‐ventilation and SPECT perfusion images in patients with airway stenosis. The basic assumption for obtaining CT‐based ventilation is that ventilation of the lung is accompanied by mechanical movement (expansion and recovery) of lung tissue, and could be captured by the 4DCT. However, other movement such as cardiac motion or anatomical changes such as pulmonary perfusion co‐exist in this process. Inevitably, the accuracy of this technique is affected by the sorting methods used to generate images for individual respiratory phases.^22^

The study performed by Mark et al.^11^ deserves special attention. The decline of pulmonary perfusion after a certain amount of radiation dose in the lung is actually consistent with the recent findings by Li et al.,^23^ in which a decrease of HP xenon‐129 gas exchanged to red blood cells was detected after radiation. This decreased amount of HP xenon‐129 gas exchange is found to be associated with increased gas exchange time and capillary transit time through MR spectroscopy. Currently, RILI is considered to be caused by direct DNA damage and reactive oxygen species (ROS) generated by the ionization of water molecules.^3^ ROS accounts for about 60% of lung damage. Blood cell death, remodeling of vasculature, and decline of perfusion are all considered to be pathophysiological changes preceding the onset of pneumonitis or fibrosis. The ability to probe these early changes throughout the radiation course will help to quantitatively identify a threshold triggering RILI and understand the mechanism of RILI. More importantly, it may provide clinicians time and opportunity to slow down or avoid some of the damage to healthy lung tissues, as illustrated in preclinical studies^24^, ^25^, ^26^

HP xenon‐129 MRI is a perfect tool for this purpose. First, it does not expose the patient to any ionizing radiation, and therefore permits repeated imaging visits. Second, HP xenon‐129 has this unique feature to investigate pulmonary ventilation, perfusion, and direct gas exchange simultaneously, which is not accessible by CT or SPECT. This is because xenon‐129, like the other xenon gas families as an anesthetic agent, has relatively high solubility in the human tissue, including pulmonary parenchyma and blood, which was called “dissolved phase”. This dissolution is accompanied by a huge chemical shift (∼200 ppm) which allows the dissolved‐phase xenon to be separated from xenon‐129 as in the gas phase using MR technique.^27^ Recent development in MR techniques allows the acquisition of three‐dimensional images of xenon‐129 in the lung parenchyma, blood and alveoli in a single breath‐hold acquisition.^16^ These features are available using other imaging tools including previously investigated hyperpolarized helium‐3 MRI applied to the radiotherapy field.^28^ In a pilot study performed by Rankine et al.,^29^ only moderate correlations were found between the measured xenon‐129 uptake by pulmonary blood and xenon‐129 ventilation in three patients with NSCLC before the radiotherapy treatment (*R *= 0.53 ± 0.02) and this correlation declined to *R* = 0.39 ± 0.07 after the treatment. Furthermore, the effective uniform dose, V_20_, V_10_, and V_5_ based on the functional information provided by xenon‐129 uptake by pulmonary blood and xenon‐129 ventilation differ by 1.5 ± 1.4 Gy, 4.1 ± 3.8%, 5.0 ± 3.8%, and 5.3 ± 3.9%, respectively. This implied the gas exchange information that can be obtained by HP xenon‐129 MRI provides a novel perspective for functional avoidance of the lung. A future extension of the work in this topic could follow two directions: First, to incorporate the option of imaging the dissolved‐phase xenon‐129 in the lung into the study; second, to apply the function‐based planning into real treatment and closely follow the lung status of treated patients. As shown in Figure 2, when the ventilation heterogeneity close to the target is high, selection of beam angle is important for dose sparing of the functional areas. With the HP xenon‐129 imaging tool, it will be very interesting to see how different regions respond to different radiation doses correspondingly and how they evolve with treatment. This will likely provide guidance information for advanced treatment planning.

There are several limitations of this study. First, a relatively small number of patients were used due to the short time span of this project. More patients will need to be investigated to further validate the findings in this study. Second, the MRI acquisition is performed in breath hold. During the radiation therapy, even the patient could be treated in breath hold, the tidal volume of the lung could still vary from the day of imaging. This will introduce dosimetric uncertainties, which exist for almost all radiation therapy procedures. Third, static IMRT is only one way to perform radiotherapy treatment planning of the lung. Three‐dimensional conformal radiation therapy (3D CRT), IMRT arc therapy are also commonly used to treat tumor in the lung.^30^ Static IMRT and IMRT arc therapy both have their pros and cons in the treatment of lung cancer.^31^ Detailed discussion about the difference between these two techniques is beyond the scope of this study. Static IMRT was chosen in this study because it allows intuitive beam angle selection to avoid the entrance of the beam through the highly ventilated lung areas and permits plan optimization to minimize dose exposure to the desired areas. In addition, we used quantitative DVH metrics including V_5Gy_, V_10Gy_, V_20Gy_, and *D*_mean_ of the lung to evaluate the treatment plan. But, whether the function‐based plan could really reduce pulmonary toxicities in patients requires validation in large‐scale clinical trials.

Lastly, the power of HP xenon‐129 MRI was not fully utilized and only ventilation information was used for plan optimization. Although an existing study^32^ showed that the same day repeatability of measuring ventilation defect using hyperpolarized‐gas MRI was relatively high for same day rescan (*R* ^2^ = 0.941) in COPD, ventilation definitely could change in a patient with both COPD and lung cancer with time.

In conclusion, in this proof‐of‐concept study, we demonstrated that function‐based radiotherapy planning based on HP xenon‐129 MRI is feasible in ten patients with NSCLC. The function‐based treatment plan was able to reduce radiation exposure in highly ventilated regions of the lung, while meeting other clinical treatment planning goals simultaneously. This finding could serve as a preparation study for a large‐scale clinical trial which makes full use of the HP xenon‐129 MRI including the capability to probe lung physiology such as gas exchange and pulmonary blood perfusion to assist radiation therapy of the lung.

## AUTHOR CONTRIBUTIONS

RT planning, data processing, and manuscript writing: Yi Ding and Lu Yang. ^129^Xe gas MRI operating and data collection: Qian Zhou, Haidong Li, Qiuchen Rao, and Xin Zhou. Data processing and assist in manuscript writing: Xiao Wang, Li Zhao, Dongsu Du, and An Liu. Treating and responsible physicians and also assist in data collection: Jianping Bi, Ying Li, Guoliang Pi, Wei Wei, and Guang Han. Experimental design and manuscript writing and final revision: Desheng Hu and Kun Qing.
